# Modulating the distribution and fate of exogenously delivered MSCs to enhance therapeutic potential: knowns and unknowns

**DOI:** 10.1186/s40635-019-0235-4

**Published:** 2019-07-25

**Authors:** Claire H. Masterson, Gerard F. Curley, John G. Laffey

**Affiliations:** 1 Regenerative Medicine Institute (REMEDI) at CÚRAM Centre for Research in Medical Devices, Biomedical Sciences Building, National University of Ireland Galway, Galway, Ireland; 2 School of Medicine, College of Medicine, Nursing and Health Sciences, National University of Ireland Galway, Galway, Ireland; 3 Department of Anaesthesia and Critical Care, Royal College of Surgeons in Ireland Education and Research Centre Smurfit Building, Beaumont Hospital, Dublin, 9 Ireland; 4 Department of Anaesthesia and Intensive Care Medicine, Galway University Hospitals, SAOLTA Hospital Group, Galway, Ireland

**Keywords:** Mesenchymal stromal cell, Biodistribution, Kinetics, Fate, Cell distribution, Cell imaging

## Abstract

Mesenchymal stem/stromal cells (MSCs) are undergoing intensive translational research for several debilitating conditions, including critical illnesses such as ARDS and sepsis. MSCs exert diverse biologic effects via their interaction with host tissues, via mechanisms that require the MSC to be in close proximity to the area of injury. Fully harnessing the therapeutic potential of advanced medicinal therapeutic products such as MSCs and their successful translation to clinical use requires a detailed understanding of MSC distribution and persistence in the injured tissues.

Key aspects include understanding MSC distribution within the body, the response of the host to MSC administration, and the ultimate fate of exogenously administered MSCs within the host. Factors affecting this interaction include the MSC tissue source, the in vitro MSC culture conditions, the route of MSC administration and the specific issues relating to the target disease state, each of which remains to be fully characterised. Understanding these factors may generate strategies to modify MSC distribution and fate that may enhance their therapeutic effect.

This review will examine our understanding of the mechanisms of action of MSCs, the early and late phase distribution kinetics of MSCs following in vivo administration, the ultimate fate of MSCs following administration and the potential importance of these MSC properties to their therapeutic effects. We will critique current cellular imaging and tracking methodologies used to track exogenous MSCs and their suitability for use in patients, discuss the insights they provide into the distribution and fate of MSCs after administration, and suggest strategies by which MSC biodistribution and fate may be modulated for therapeutic effect and clinical use.

In conclusion, a better understanding of patterns of biodistribution and of the fate of MSCs will add important additional safety data regarding MSCs, address regulatory requirements, and may uncover strategies to increase the distribution and/or persistence of MSC at the sites of injury, potentially increasing their therapeutic potential for multiple disorders.

## Background

Mesenchymal stem/stromal cells (MSCs) were first described in the 1970s as a clonogenic fibroblast precursor cell population of the bone marrow, referred to at the time as ‘colony forming unit fibroblasts’ [1, 2]. These cells were plastic adherent, had a fibroblast-like morphology, could be differentiated down chondrogenic, osteogenic and adipogenic lineages [3], and continuously expanded ex vivo [4]. MSCs can now be isolated from other tissue sources including adipose and placental tissues. Current MSCs identification strategies utilise their expression of cell surface markers, and their ability to differentiate down mesoderm (chondrogenic, osteogenic, adipogenic) lineages [3]. MSCs are positive for CD105, CD73, CD44, CD90 and negative for CD45, CD34, CD14 (i.e. haematopoietic markers), CD11b, CD79α/CD19 and HLA-DR [3].

Unfortunately, for a number of disease targets, promising results demonstrated in diverse pre-clinical models have to date largely not successfully translated in subsequent phase 2–3 clinical trials [5]. Understanding the reasons underlying prior translational failures for cellular therapies may improve the likelihood of subsequent success, including for critical illnesses such as sepsis [6] and ARDS [7]. A significant knowledge gap has been the understanding of the biodistribution of MSCs following exogenous administration, and of their ultimate fate within the host.

A better understanding of patterns of biodistribution and of the fate of MSCs will uncover strategies to enhance strategic targeting and/or persistence of MSC at the sites of injury, potentially increasing their therapeutic ability. However, determining the biodistribution and fate of MSCs is clearly more challenging than for a standard pharmacologic. In this regard, recent advances in imaging techniques now offer the possibility to more systematically develop our understanding of these issues.

Fully harnessing the therapeutic potential of advanced medicinal therapeutic products such as MSCs and their successful translation to clinical use requires a detailed understanding of MSC distribution and persistence in the injured tissues. This review will examine our understanding of the mechanisms of action of MSCs, the kinetics and dynamics of MSCs in vivo, and the potential importance of these to mediating the effects of MSCs. We will critique the cellular imaging and tracking methodologies currently used to track exogenous MSCs, discuss the insights they provide into the distribution and fate of MSCs after administration, and suggest strategies by which MSC biodistribution and fate may be modulated for therapeutic effect.

## MSCs mechanisms of action

MSCs exhibit a diverse array of effects and multifunctional mechanism of action, with no single, overarching biological effect. The precise mechanisms by which MSCs modulate the injury or reparative processes appear to depend on the specific injury microenvironment and the pathobiology of the injurious/repair process itself. MSCs appear to act predominantly by modulating the host response, both directly and indirectly, with the interaction with the immune system of central importance.

### ‘Contact-dependent’ mechanisms

These mechanisms of action comprise those that necessitate the presence of MSCs at or near the sites of injury [8]. Islam and colleagues demonstrated a ‘contact-dependent’ mechanism of action whereby MSCs were shown to directly contact and transfer cellular components to lung epithelial cells [8]. Key elements of MSC-macrophage interactions may also be contact-dependent [9]. A recent study has shown the transfer of MSC extracellular vesicles to macrophages via tunnelling nanotubules [10, 11]. The capacity of MSCs to migrate or ‘home’ to sites of injury, a key characteristic that may affect the efficacy of contact-dependent mechanisms of action, may be enhanced for therapeutic benefit. The migratory capacity of MSCs can be modulated by factors such as culture, passage number, donor age and the dosage of MSCs [12].

### Paracrine mechanisms

Mediators secreted by MSCs also mediate multiple effects. The MSC ‘secretome’ has been shown to mediate, at least in part, repair following ventilator-induced lung injury and to enhance bacterial killing in a number of studies [13–16]. The MSC secretome is a complex array of components, ranging from soluble secreted factors to factors encapsulated in extracellular vesicles. Extracellular vesicles range in size, composition, contents, and quantity, with different properties depending on source and method of release (due to stress, inflammation or other cues) [17]. The transfer of MSC exosomes containing miRNAs to endothelial cells have been shown to promote angiogenesis in HUVECs both in vitro and in vivo [18], and the production of hepatocyte growth factor by MSCs has been shown to be responsible for the restoration of endothelial monolayer integrity following LPS-induced permeability [19].

### Trans-differentiation

While trans-differentiation is not considered a major mechanism of action, a small number of studies have demonstrated the in vivo differentiation capacity of MSCs after transplant. One such study demonstrated that MSCs engrafted and differentiated to AEC II cells in the lung, and their behaviour was influenced by the injurious environment [20]. Cells were detected up to 28 days post-transplant using PCR and histopathology.

## Optimising the therapeutic potential of MSCs

Strategies to optimise MSC efficacy have the overarching aim of developing a more potent, efficient MSC therapeutic, thereby producing both a more effective therapy and/or at a reduced dose of administered cells. A successful optimisation strategy would reduce production costs and potentially be safer for patients.

### Optimising culture conditions

Following isolation of MSCs, optimal culture conditions are essential to maintain therapeutic potential. MSCs reside in hypoxic environments in vivo and exposure to normoxic conditions (21% O2) leads to oxidant generation, premature senescence, loss of ‘stemness’ [21] and a reduction in proliferation capacity [22]. MSCs from older mice had an age-related decrease in receptor and cytokine expression important for migration and activation of MSCs in response to inflammation, suggesting that donor age is critical to efficacy [23, 24]. Prolonged culture and passage of MSCs leads to lower levels of HLA-DR expression than when the cells are initially cultured [25]. MSC cryopreservation, which is important in making larger-scale multi-centre studies feasible, and ultimately for use in the clinical setting, may reduce MSC potency and viability [26].

### Activation of MSCs

Strategies to ‘prime’ MSCs before administration aim to further enhance their therapeutic potential. These strategies range from alteration of culture conditions to exposure of MSCs to inflammatory cytokines to mimic the injury microenvironment and induce a pre-activated phenotype [27–29]. An alternative approach is the use of gene overexpression strategies to enhance MSC production of key effector proteins [30–33].

### Biodistribution and fate of MSCs

MSCs mediate their effects either by direct cell-cell contact, by the secretion of mediators that exert a paracrine effect on nearby cells and tissues, and (perhaps) via in situ trans-differentiation to directly replace injured cells. Consequently, exogenously administered MSCs need to become distributed within the host such that they are either in or in very close proximity to the injury site in order to exert their effects. This issue is underlined by the fact that the quantities of molecules produced by the current administered doses of MSC are vastly less than would be administered when using other biologics (picogram vs milligram range) [34].

Altering MSC distribution patterns within the host—termed ‘biodistribution’—is an important, but poorly understood, strategy by which MSC efficacy may be enhanced. In addition, prolongation of the contact time between MSCs and the injury site may also enhance the efficacy of MSCs. To date, studies carried out to assess therapeutic efficacy rarely assess the pattern and/or impact of MSC biodistribution, and likewise studies carried out to determine exogenous MSC biodistribution within the host do not assess their efficacy.

## MSC biodistribution: current insights

**Fig. 1:**
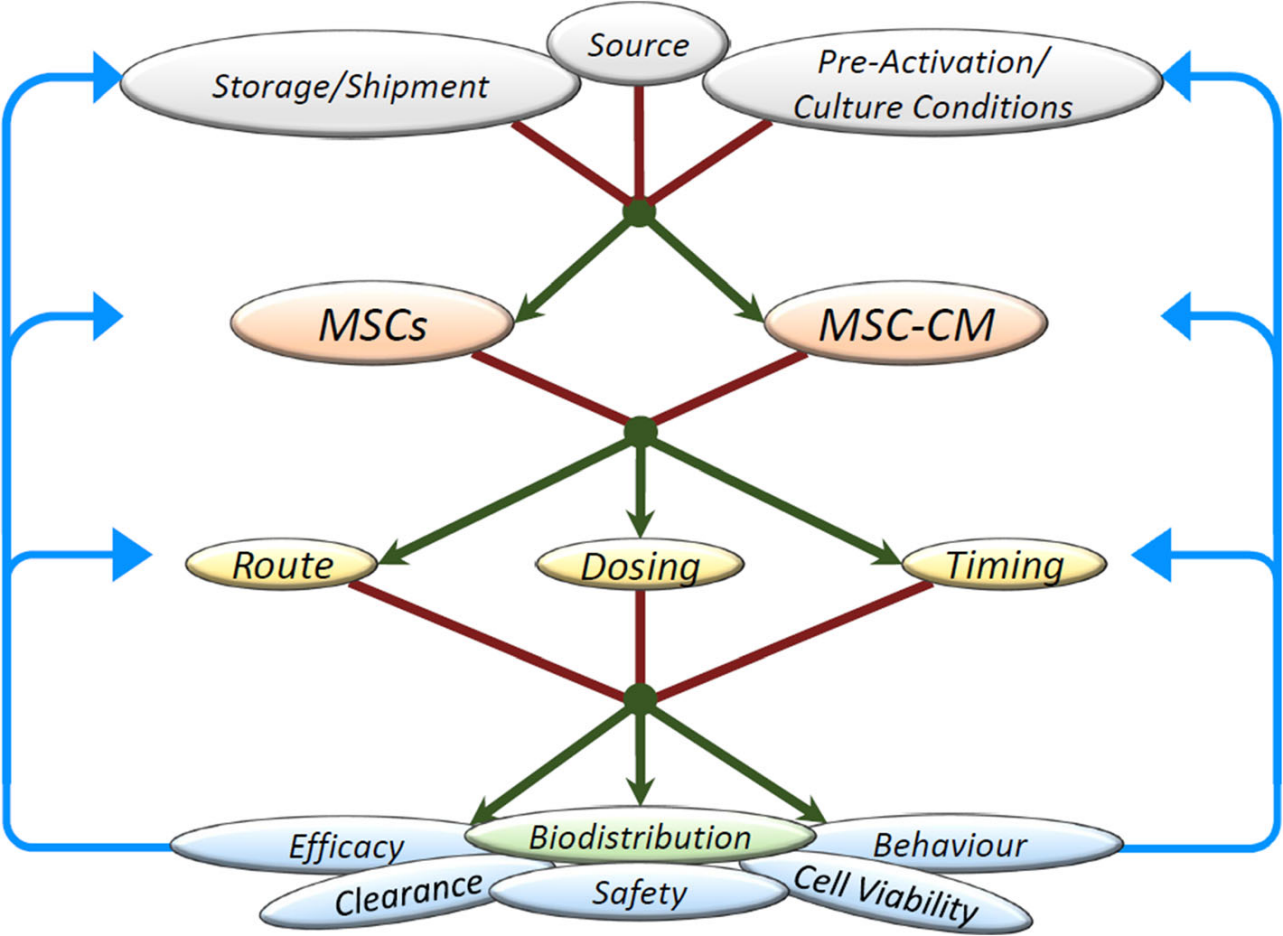
Schematic representation of the factors which influence cell biodistribution in vivo. The source of MSCs (mainly adipose, cord or marrow), culture conditions, pre-activation strategies and the method of storage before administration influences the quantity and quality of MSCs and conditioned media (MSC-CM) produced. The decision to use either the MSCs themselves or MSC-CM, as well as considering the condition to be treated, will influence the chosen route, dosage and timing of administration which will in turn influence the biodistribution pattern of the cells or cell products. Biodistribution data will furnish knowledge of the safety, efficacy, viability, behaviour and clearance rate which will feedback to the optimal source, culture, storage, route, dosage and timing strategies

Multiple factors affect the biodistribution of systemically administered MSCs (Fig. 1). MSCs range in size from 20 to 60 μm and become physically obstructed in the microcirculation of organs, which measure 5–10 μm in diameter. Systemically administered MSCs must traverse the lung vasculature, with a significant proportion of the administered MSCs becoming ‘trapped’ in the lung shortly after administration. MSCs can remain in the pulmonary tissues for up to 24 h after which they were not detected [35]. This is a significant impediment to therapeutic efficacy for MSCs administered for systemic conditions, given the need for MSCs to be in close proximity to the site of injury. Several studies have examined the use of local administration (discussed below) or other methods to bypass the pulmonary capillary bed such as vasodilators [36] or intraarterial delivery [37]. However, systemic administration is a more desirable, less invasive method for treatment of organs such as the heart and brain, and the ease of access and proof of efficacy in several pre-clinical MSC-treated models make it the most likely route to be used in the majority of studies.

Nystedt and colleagues have also demonstrated a role for cellular interactions mediating MSC retention in the lungs [23]. They reported that umbilical cord-derived MSCs had a faster lung clearance rate than bone marrow-derived MSCs (BM-MSCs). In addition, the surface adhesion marker profile differed between the cell types, and with increasing BM-MSC donor age. Cells which were trapped in the lungs after administration were shown by histological examination to be localised to the endothelium with movement to the lung stroma observed at 24 h [23]. Other studies have observed intravascular rolling and adhesion of MSCs involving the adhesion molecules VCAM-1/VLA-4 and P-selectin after intraarterial injection [38]. Intravital microscopy experiments have demonstrated the transmigration of MSCs between and through endothelial cells in the dermal microcirculation in a similar but slower process than leukocytes [39]. MSCs have been shown to migrate to the liver, kidney and/or spleen after clearance from the lung; however, detection methods that determined this generally did not ascertain if the signal obtained was from viable, intact MSCs, from cell fragments or free label. The type and location of tissue injury may alter MSC distribution. Systemically delivered MSC to models of myocardial infarction was shown to differentially distribute between healthy and injury models with increased numbers of radiolabelled cells detected in the infarcted heart after administration [40].

In summary, a better understanding of patterns of biodistribution and of the fate of MSCs will uncover strategies to increase the distribution and/or persistence of MSC at the sites of injury, potentially increasing their therapeutic potential for multiple disorders, including critical illnesses such as ARDS and sepsis.

## MSC fate: current insights

Intravenously administered MSCs first accumulate in the lung vasculature, move to other major organs such as the liver and kidneys and after a variable but short period (24 h to 14 days) are no longer detectable in the body. The ultimate fate of exogenously administered MSCs remains unclear. While reports of MSC engraftment and differentiation exist, the numbers detected are vastly lower than the administered number of cells. There are further reports of MSCs being phagocytosed by the host immune system as a possible mechanism of their clearance [41]. Another potential method of MSC clearance is that MSCs under stress may undergo apoptosis and break up into micro-particles. This mechanism first suggested following in vitro experiments by Bian et al. [42]. These microparticles could then be redistributed around the body to be either phagocytosed or filtered by the kidneys for excretion.

The host immune system plays a role in MSC clearance. While traditionally thought to be ‘immune-privileged’, it appears that MSCs are better characterised as ‘immune-evasive’ [43]. Studies demonstrate the production of antibodies against MSC transplant and rejection of same [44–46] albeit in a slower manner than rejection of other allogenic cell types such as fibroblasts [43]. The host can acquire immunity to MSCs after multiple dosing by an increase in the percentage of memory T cells after administration [47]. In this regard, in mice that are T and B cell, or NK cell deficient, and in irradiated mice, MSCs were shown to persist longer than in immunocompetent mice [48].

Cryopreservation of MSC, which facilitates storage for clinical use, may increase MSC clearance in vivo. MSCs which were thawed and administered via intramuscular injection were cleared within 3 days as compared to those grown in culture overnight which persisted for up to 3 months [49]. These findings support a previous study demonstrating cytoskeletal disruption and differences in biodistribution between fresh and frozen cells [50].

Perhaps most intriguingly, in terms of MSC fate, is the recent demonstration by Galleu and colleagues that MSC apoptosis after administration may be necessary for therapeutic benefit [51]. Patients with graft-vs-host disease that had a more cytotoxic response to MSCs also responded better to the MSC therapy.

In summary, our understanding of the fate of exogenously administered MSCs, a potentially central issue in regard to maximising the efficacy of MSCs, remains a significant knowledge gap.

## Methods of imaging MSC distribution and fate

**Fig. 2:**
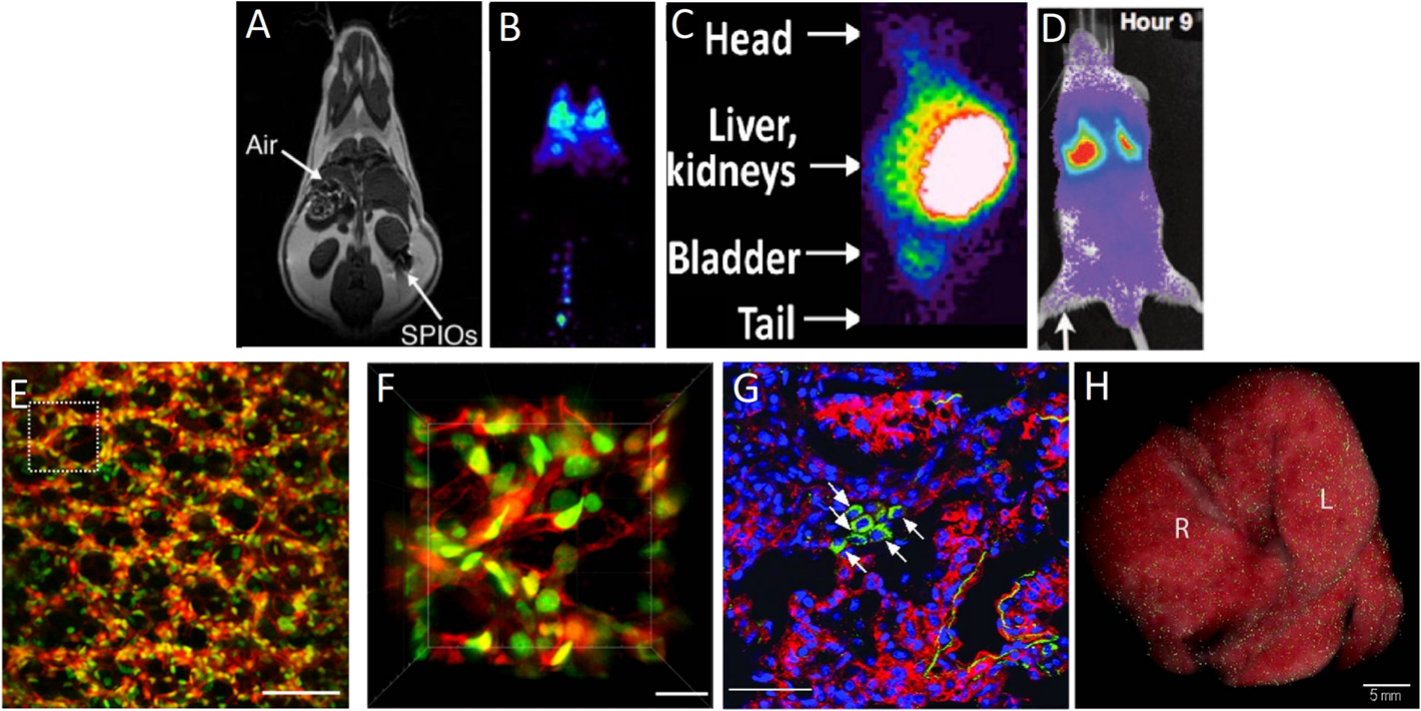
A comparison of the imaging techniques for analysing biodistribution. **a** MRI scan revealing detailed anatomical structure but poor distinction between air-tissue interface and SPIO radiolabel [123]. **b** PET detection of IV administered radiolabelled MSCs showing good resolution with no anatomical structure [124]. **c** Gamma camera acquisition of radiolabelled IV administered cells demonstrating lower resolution than PET and no anatomical structure [125]. **d** BLI detection of luciferase reporter-labelled MSCs administered IV demonstrating a lower sensitivity than PET/SPECT and external anatomical structure alluding to in vivo cell location [126]. **e**, **f** Intravital microscopy imaging of live tissues demonstrating the specificity of labelling at the cellular level (pulmonary tissue; cytoplasm = red, nuclei = green) [127]. **g** GFP-labelled MSCs detected in lung tissue sections (green) with cell nuclei clearly visible (blue) [128]. **h** The use of the CryoViz® system offers the 3D reconstruction of histological sections containing MSCs labelled using Q dots [71]

The ideal imaging technique would incorporate a label/probe which is non-toxic, that does not alter the cell morphology or phenotype, and is reliable in terms of labelling method, signal strength, and is long-lived in vivo (Fig. 2). A desirable quality would also be that the label/probe would not be detectable upon administered cell death. The detection method should be non-invasive, not cause harm to the subject, be rapid and sensitive, and be capable of repeated use. A further desirable quality would be that it could render 3D images and display anatomical structures as well as relaying real-time events in cell tracking. For observing MSC interactions in vivo, sensitivity at a micron range would be essential.

Current techniques fulfil the majority of these properties separately (Table 1). However the main obstacle to their routine clinical use is the use of probes, labels, and detection methods which may compromise either the administered cell’s function or the host themselves. Many of the studied detection methods are not sensitive enough without being invasive or having the need for post-mortem samples. Previously, methods of cell tracking in vivo were limited to post-mortem analysis of excised and sectioned tissues, limiting time points and requiring sacrifice of multiple animals. Advances in whole body, vital in vivo imaging have led to the use of systems such as MRI, PET and SPECT and specialised small animal systems such as in vivo imaging systems (IVIS) using near-infrared (NIR). The following paragraphs discuss imaging/tracking methodologies used, their principles, suitability, limitations and possible translation to clinical situations.

**Table 1:** A comparison of methods used to analyse MSC biodistribution patterns in animal models

Method	Used clinically	Terminal/post-mortem	Single-cell resolution	Benefits	Limitations
MRI/CT	✓	X	X	Good anatomical structure, unlimited depth, can be used with PET/SPECT	Poor distinction between probe and air, possibility of free probe, expensive, low sensitivity
PET	✓	X	X	High resolution, unlimited depth	Expensive, shorter half-life of probe, no anatomical data, radiation exposure
SPECT	✓	X	X	Inexpensive, longer-lived probe, unlimited depth	Low resolution, no anatomical data, radiation exposure
BLI	X	X	✓	Can distinguish live/dead cells, inexpensive, high sensitivity	Poor tissue penetration, single-cell distinction not possible
PCR	✓	✓	X	Highly specific, widely available	Limited observational area, cannot distinguish live/dead, detects genetic material only
Histology	✓	✓	✓	Accessible technique, high-quality images obtained, can demonstrate cell viability, interactions, molecular changes	Cannot account for changes during processing, limited observational area. Requires biopsy/post-mortem samples
Intravital microscopy	X	✓	✓	Highly sensitive, allows observation at micrometre scale, phenotypical and morphological cell changes observable	Highly invasive, requires specialised techniques/equipment, limited observational area

### Labelling of MSCs

The purpose of cell labelling is to distinguish administered cells from host tissue and to easily identify their movement and behaviour in vivo. Ideally, cells should be easily detectable to a high-resolution over long periods, be unchanged from their naïve phenotypical and morphological state, be non-toxic and be located using a minimally invasive method. There are several methods of cell labelling, and developments in recent years have allowed higher resolution, more accurate and long-term analyses of their journey in vivo.

#### Fluorescent and bioluminescent labelling

MSCs can be fluorescently labelled using fluorophores linked to a specific molecule in/on the target cells or transduced with a bioluminescent or fluorescent protein reporter gene. These labelling methods are well characterised and commercially available requiring an ex vivo preparation (transduction or transfection) of the cells before administration, allowing for characterisation of phenotypical and morphological properties and toxicity beforehand. Detection of fluorescence labels after administration requires the application of an excitation light source at specific wavelengths to activate the emission of fluorescent light from a fluorophore. Bioluminescent labelling generally requires the administration of probes (substrates) which are catalysed by the intracellular enzyme (generally luciferin or coelenterazine produced by transduced reporter genes) to produce a bioluminescence signal [52]. Difficulties occur due to the limitations arising from the ability to detect fluorescent/bioluminescent cells in deeper tissues via whole body imaging. Developments in microscopy techniques such as multiphoton excitation which allows high-resolution image acquisitions in thicker tissue sections than conventional fluorescence microscopy [53] have overcome this challenge to some extent. Near-infrared (NIR) imaging is emerging as a promising in vivo detection method. With the development of safe and effective contrast agents and the use of fluorescent nanoprobes, NIR provides the ability to penetrate into the deeper tissues [54–56].

#### Labelling using radionucleotides

The use of radionucleotides is a currently approved method in clinical use allowing for a non-invasive detection of labelled cells and tissues [52]. Cells can be exposed to labelling agents which are extracellular—binding the cell membrane via linking to lipophilic long chain esters, such as ^64^Cu-DOTA-HB, or are intracellular following passive or transporter uptake such as ^111^In-oxine or ^18^F-FDG, and are detected by SPECT or PET imaging in vivo [57]. An indirect method of radionucleotide labelling involves the use of reporter gene constructs in MSCs which produce proteins with a high affinity for radionucleotides which can be injected separately at multiple time-points [58]. This overcomes some of the shortfalls of this method due to the generally short half-life of the particles. SPECT and PET are often used in conjunction with MRI or CT, which uses superparamagnetic iron oxide (SPIO)- or AuNPS-based contrast agents to confirm the anatomical location of labelled cells using image overlay. Indeed, SPIO or AuNPS can be directly used as intracellular MSC labels as they are taken up by endocytosis ex vivo [57], although the sensitivity of these detection methods can be poor [58].

#### Benefits, limitations and insights

The benefits of using fluorescent labels are that the effects on the cells are well characterised, multiple coloured dyes can be used and easily distinguished using different excitation/emission wavelengths, and in certain cases it will allow for the distinction between dead and viable cells in vivo. The use of reporter genes can also demonstrate the expression of certain transgenes in response to the in vivo environment giving further insight to the in vivo behaviour of MSCs [59, 60]. There is a concern in the use of DNA-binding dyes such as DAPI and the introduction of reporter constructs using viral vectors which may have some downstream immunogenicity or mutagenesis potential, making them unsuitable for clinical use [61]. Methods of detecting fluorescence often require ex vivo analysis of tissues or terminal in vivo techniques eliminating the possibility of reusing animals at different time-points. This can be overcome by using whole body imaging techniques such as PET and SPECT. The use of radiolabelling methods such as ^111^In-oxine has been reported as a suitable, safe method of MSC tracking in human subjects; however, tracer leakage has been reported from MSCs [62]. It has been also reported that prolonged storage of radiolabelled (^111^In-oxine) leukocytes leads to diminished chemotaxis, high spontaneous release of radionucleotide and impairs cellular function [63, 64]. However, the majority of radioactive probes used in recent pre-clinical studies are FDA- and EMA-approved for clinical use [58]. Limitations to the use of radionucleotide/contrast agent labels include a short half-life and ‘bleaching’, difficulties in tracking cells in real-time and over longer periods in the same animals, unclear distinctions between labelled cells, free label and phagocytes containing cell particles, and depending on the method of labelling, there may be an impact on cell viability. Reporter construct labelling, which produces a high-affinity protein, can overcome the issue of radio label longevity by persistent gene expression for the duration of the cell life-span [58].

### Real-time PCR

#### Principle

Real-time or quantitative PCR (qPCR) has been used for in vivo tracking due to the ability to detect on a genomic level the relative amounts of integrated particles based on their gene expression. In a tissue sample, it would therefore be advantageous if one could discriminate between host and administered cells in terms of MSC administration. To do so using qPCR, it is necessary to identify a target for quantification. Researchers have used various techniques, the most common being to transduce or transfect a reporter gene such as luciferase or GFP into the cells before administration to allow for definitive identification by gene expression. Other methods involve the use of male donor cells (XY) into a female recipient (XX) and subsequent detection of the Y chromosome, or human cells into other species and subsequent detection of human-specific genes.

#### Studies performed

Devine and colleagues utilised GFP-transduced MSCs in their biodistribution study in immunocompromised baboon models. Analysis of distribution was performed by real-time PCR analysis of eGFP cDNA content in various tissues between 9 and 21 months post-infusion [65]. Development of qPCR assays for the detection of male, murine and human MSCs after infusion to mice allowed the detection of DNA from MSCs up to 300 days post-implant in the central nervous system of neonatal and adult mice [66] demonstrating the benefits of qPCR as a detection method over extended periods of time. Post-mortem samples from 15 patients who received MSCs were examined by PCR for engraftment in various tissues [67]. In these patients, MSC DNA was detected in samples of 8 of the patients with an overall pattern of limited engraftment, without ectopic tissue formation. Furthermore, MSC treatment response did not correlate with engraftment rates.

#### Benefits, limitations and insights

Real-time PCR as a method of detection of MSCs requires the harvest of tissues for RNA isolation. Therefore, a limitation of this method is that animals need to be sacrificed at various time-points for a complete observation of distribution over time. The potential for biopsies to render a true representation of the biodistribution pattern, given that only a small segment of the organ can be analysed, is unclear. This approach does not distinguish between MSC cellular contents delivered by intact cells or from fragments or vesicles from MSCs. However, this technique may demonstrate that the contents of administered MSCs travel beyond the lungs and engraft in other organs over long periods of time [65]. In other words, qPCR will not distinguish between viable, intact MSCs and free or integrated target sequences. The benefits of using qPCR as a detection method are that it is highly specific and depending on the target gene chosen (e.g. sex-linked chromosome gene mismatches) and there may be no need to label the cells before administration. The high specificity of this method means that only a small sample of tissue is required for detection, such as patient biopsy samples [68, 69].

### Ex vivo imaging of tissues

#### Principle

In this approach, following in vivo administration of fluorescently labelled MSCs, the tissues of interest are excised post-mortem, fixed and sectioned for imaging using fluorescence microscopy. This method has been used successfully in mouse models of corneal injury who received GFP- or Q-dot-labelled MSCs [70] using fluorescence microscopy of fixed and sectioned specimens. Using methods such as the CryoViz® imaging system a 3D reconstruction of whole organs/small animals can be amalgamated [71]. The CryoViz® system is used to create serial sections of whole organs or even whole small animals, acquire images of each section using brightfield and confocal microscopy, and reconstruct the whole organ/animal as a 3D output. Flow cytometry has also been used to detect MSCs in the peripheral blood [70] and tissue homogenates [13] at various time points following injury.

#### Studies performed

Fluorescent protein labelling or reporter gene transduction followed by histology and/or immunohistochemistry are the most common procedures in these studies [72, 73]. Studies have also analysed tissues ex vivo using fluorescent antibodies against the administered human cells in rat models of brain injury [74]. Fluorescently labelled MSCs were injected into models of corneal injury and subsequently detected locally using epifluorescence microscopy in ex vivo corneas, and in the circulation by flow cytometry [70]. The study found that increased levels of substance P and SDF-1 occurred at the injury site and suggested their responsibility for increased MSC homing to the injury. The researchers also found that MSCs persisted in the cornea up to 50 days post-injection. Using the CryoViz® system, Schmuck and colleagues performed a biodistribution study after intrajugular administration to a rat model of lung injury [71]. Between 60 min and 240 min post-administration over 99% of the detected cells were found in the lungs, liver, and spleen—the liver was the primary site of accumulation in this study.

#### Benefits, limitations and insights

The ex vivo imaging of tissues requires the sacrifice of animals at various time points and limits the analyses to the tissues excised and the area processed for imaging. The use of detection methods such as CryoViz® reduces the workload of isolation and processing and can be performed on whole organs and indeed whole mice to give an overall perspective of cell biodistribution. The chosen label will have an impact on the readout of the experiment. For example, DAPI-labelled cells administered to mouse models of myocardial infarction were detected 7 days after infusion in infarcted areas of the heart [40] by observation of tissue sections post-mortem. DAPI staining provides information on the presence of the MSC nuclear material, but not in the size, intactness or morphology of the MSC.

Flow cytometry can be performed on blood samples to detect circulating MSCs or using tissue homogenates to detect tissue-resident cells. Again, this generally requires post-mortem analyses and a requirement for many animals at different time points. Flow cytometry analyses will allow for quantification of cell number per gram of tissue and can discriminate live and dead cells [75]. Further analyses of the cells can be performed using FACS analysis alluding to their phenotypical changes in vivo.

### In vivo imaging systems

#### Intravital microscopy

Intravital microscopy (IVM) involves the use of fluorescent microscopy directly over the area of interest in live animal models, usually of the visible vasculature close to the surface of the tissue/organ under study. The passage of fluorescently labelled molecules and cells can then be recorded in real time allowing for the calculation of particle kinetics in a live model. The use of microscopy and the advances in cell-labelling techniques mean that observation on a micron scale is possible. This allows visualisation of interactions and behaviours of the MSCs such as rolling and adhesion, transmigration, velocity within blood vessels and interactions with other cell types that would not be possible using other techniques.

##### Studies performed

IVM has been used to study a range of in vivo mechanisms including glomerular filtration in the kidneys [76], pancreatic blood flow [77], cancer cell motility [78], and immune cell trafficking (reviewed in [79]). IVM can be used to image fluorescently labelled cells in vivo by the application of a fluorescent microscope lens directly to the area of interest [80–83]. This allows the real-time visualisation of the transit of systemically and locally applied fluorescent cells within the tissue and vasculature and observation over a limited period of time and their behaviour and interactions in live tissues. The transit of MSCs in a model of dermal inflammation [39] or adhesion molecule p-selectin knockout mice [38] was examined by visualisation of the vasculature in the ear. These studies allowed the visualisation in real time of the interactions of MSCs with the vasculature and the effects of resident blood cells. Intravital microscopy has also been used to observe MSCs ‘in transit’ in the vasculature of cremaster muscles allowing visualisation of MSC longevity, velocity, and deformability [84].

##### Benefits, limitations and insights

The ability to visualise both the transit and interaction of MSCs in a live animal model provides significant insights into mechanisms of action. IVM allows the visualisation of individual cells and even molecular interactions in vivo, a level of magnification that cannot be achieved currently in most other methods used. IVM is mainly constrained by the range of tissue penetration which is limited to the first 100 μm below the surface [85] but can be extended up to 1 mm (depending on tissue imaged) using multiphoton microscopy [79]. The observational area during the procedure is also quite small with generally a window of approximately 10 mm available over the site of interest. Depending on the area of interest, the invasiveness of the surgery, and the prolonged period under anaesthesia, the animal generally has to be sacrificed at the end of the procedure. This may only allow a constrained period of observation and multiple animals would be required for longer studies. However, the tissues from these animals can be retained for subsequent histological analyses.

### Whole body imaging

#### Principle

Whole body imaging can be a challenging technique whereby one needs to consider a range of factors. In the context of tracking the biodistribution of individual cells, careful consideration needs to be given to the probe used to distinguish the administered cells from other cells and tissues of the body. One immediate difficulty is apparent—the need for a probe which will be strong enough for detection through the skin (and fur in pre-clinical models) and a detection method which will give the precise anatomical location and a sensitivity to detect individual cells. The use of radiolabelled cells is a popular choice for whole body imaging due to both their routine use in the clinic and the strength of the signal produced. Whole body tomography is a useful tool that allows for real-time tracking of radiolabelled cells at multiple time-points without tissue biopsy or animal sacrifice or injury. Positron emission or single photon emission computed tomography (PET or SPECT) are the two main methods of detection of radiolabelled cells and can be used in combination with X-ray CT or MRI to show the exact location in vivo [58].

Dynamic near-infrared fluorescence (DNIF) has been investigated for fluorescent probe detection in human subjects with promising results [86]. Using an IV bolus of indocyanine green, DNIF could detect the passage of the administered probe in a whole body image rendering images to show the real-time transit in the body. Luminescence has been generally considered a poor candidate for whole body imaging due to its weak signal and pseudo-colour-generated images [87] and the need for circulating luciferin [88]. NIR persistent luminescence nanoparticles have been developed recently [89] and are considered a promising candidate for whole body bioimaging due to the persistence of luminescence which can last for hours to days after excitation [90]. The development of the in vivo imaging system (IVIS®) in recent years has allowed deeper penetration of tissues for the detection of bioluminescence [75].

#### Studies performed

Earlier studies of administered bone marrow MSCs used techniques such as radiolabelled cells administered intravenously (IV) or intraarticularly (IA) followed by whole body computed tomography (CT) scans [37, 91, 92]. The majority of these studies concluded that systemic infusion of MSCs resulted in an initial accumulation in the lung [35, 93]. One of these studies noted that the use of vasodilators encouraged MSC clearance from the lungs indicating the role of cell size versus capillary diameter in lung accumulation [93]. A more recent study has examined MSC distribution in an uninjured porcine model using radiolabelled cells administered either IV or IA [94]. CT scans revealed the accumulation of cells in the lungs, kidneys, liver, spleen and vertebrae in descending order. IA administration resulted in a reduced lung accumulation due to a bypass of the pulmonary capillary bed. Neural MSCs were transfected with ^111^In labelled silica nanoparticles and administered via intracranial or intracardiac injection to mouse models of glioblastoma [95]. Mice were imaged using SPECT/CT demonstrating that labelled neural MSCs migrated toward the tumour site after systemic administration. In patients with advanced liver cirrhosis, radiolabelled MSCs were administered systemically and patients subjected to planar whole body image acquisitions at various time-points thereafter [96]. The study demonstrated MSC accumulation initially in the lungs, followed by increases in the spleen and liver up to 10 days post-administration. MSC labelled with near-infrared fluorescent nanoparticles were administered to mouse models of Chagas disease and visualised using an IVIS® in combination with PET scans [97]. More recently, Cao and colleagues performed a study using bioluminescence and the IVIS system both in vivo and ex vivo demonstrating migration of the MSCs in healthy animals to the lungs, kidneys and lower back and complete MSC clearance from the animal by day 14 [75]. Bioluminescence has also been used in animals subjected to colitis injury and intraperitoneal (IP) administration of MSCs [98] demonstrating that distribution of MSCs was largely dependent on the route of administration rather than the inflammatory environment. Further analyses by this group deduced that MSC presence at the site of inflammation was necessary for therapeutic effect.

#### Benefits, limitations and insights

Use of MRI or X-ray CT scans to detect labelled cells harbours little to no risk to the subject. However, the effect of multiple scans is not known but assumed to be low risk [99, 100]. Although PET and SPECT imaging are high sensitivity imaging modalities which can detect radioisotopes on a picomolar scale [58], these techniques do not have a single-cell resolution. Isotopes used in PET scanning have a shorter half-life than those used in SPECT scanning (up to 4 days vs up to 8 days). This can be overcome by using a combination method with a reporter gene construct as mentioned previously [58]. The key limitation to PET/SPECT/MRI methods of detection is that there can be no distinction between viable, intact cells and free or phagocyte-engulfed label. PET imaging allows detection at a higher sensitivity compared to MRI but a lower spatial resolution and no anatomical data to pinpoint location with both methods resulting in lower resolution imaging at cellular and sub-cellular levels [56].

### Strategies to alter MSC biodistribution and fate

#### Decreasing MSC lung trapping

The co-administration of the vasodilator sodium nitroprusside can enhance MSC passage through lung capillaries [101]. Inhibition of CD49d may also facilitate passage through the lung, while administration of the MSCs in divided doses may also aid trans-pulmonary passage [102]. Intrahepatic arterial MSC injection in a mouse enabled bypass of the lung vasculature, with up to four times more cells accumulating in the liver compared to standard IV injection [75]. However, a previous study reported the formation of pulmonary emboli after intraarterial administration of MSCs in mice [103] suggesting the need for care with this route.

#### Local delivery of MSCs

Local MSC delivery may result in the accumulation and persistence of substantially higher numbers of MSCs at the site of injury. Intracoronary injection resulted in a significant retention of cells in the cardiac tissues compared to peripheral vein administration [104]. However, intracoronary delivery may cause decreased coronary blood flow [105], and even acute MI [106], suggesting the need for caution. Radioactively labelled MSCs were injected either IV or into the left ventricular cavity of rats subjected to myocardial infarction. The intraventricular administration of MSCs was demonstrated to overcome the accumulation of MSCs in the lung seen after IV administration [91].

Iron oxide nanoparticle-containing MSCs were administered either IV or directly into the corpus callosum of healthy and LPS-injured rats and detected by MRI up to 30 days post-administration [107]. Locally administered cells in healthy animals remained localised to the administration site and migrated toward the lesion in injured animals after both IV and IC administration. In healthy pigs, IA administration of radiolabelled MSCs resulted in a lower accumulation in the lungs and a higher MSC content in the liver after 8 h as demonstrated by SPECT/CT imaging [94].

An interesting study by Chen and colleagues [108] examined the differential migration properties of MSCs after injection into the bone marrow or caudal vein of mice with a liver injury. Using PCR, flow cytometry and cryosectioning, it was determined that liver injury mobilised the transplanted MSCs from the marrow toward the liver, a mechanism dependent on cytokine release from the injury site. A comparison of delivery routes was performed using IV, IP, SC and IM in healthy animal models, and detection of these cells was carried out using bioluminescence detection [49]. IV MSCs were undetectable within days, IP and SC persisted for up to 4 weeks, but IM injection resulted in MSC detection over 5 months.

#### Production of smaller MSCs

In contrast, culture conditions that result in the production of smaller MSCs may significantly alter biodistribution by reducing MSC and size obstruction trapping in capillary beds of the lung. Ge and colleagues [109] have compared different culture methods: 3D hanging drop vs 2D monolayer culture and demonstrated that MSC size was much more homogeneous and smaller in 3D culture with an average cell diameter of 12.6 μm vs 26.5 μm from the monolayers. Gene overexpression approaches may facilitate targeting of MSCs to specific targets. It has also been shown that the endothelial-binding molecules on the surface of MSCs can be altered ex vivo affecting their binding ability after administration [110–113].

#### Enhancing MSC migration

Enhancing the migration potential of MSCs in vivo may increase their presence at the site of injury. Overexpressing receptors such as CXCR4 which mediate migration of MSCs could facilitate the enhancement of the cells in vivo as demonstrated by Yang and colleagues [114]. The CXCR4 receptor expression is reduced in cultured MSCs over time [115] and by overexpression of CXCR4, Yang and colleagues demonstrated an enhanced migration potential in mouse models of ALI 2 weeks after transplant [114]. In a study to compare MSC migration capacity toward 16 different growth factors and chemokines, it was shown that untreated MSCs migrate differentially than MSCs pre-treated with TNF-α which have a greater migration capacity [116].

#### Increasing MSC longevity

This strategy is based on the contention that increased MSC longevity and/or retention at the injury site may enhance effectiveness. Melatonin has been examined as a potential treatment to induce longevity and ‘stemness’ of MSCs [117] and the use of these pre-treated MSCs in vivo increased their survival and reduced apoptosis in cerebral ischemia [118]. Transducing MSCs with lentiviral vectors to induce COX1 overexpression enhanced the therapeutic efficacy of the cells in pulmonary hypertension models and also increased their longevity to at least 21 days in the lung tissue [119].

#### Altering MSC cell surface markers

The cell surface molecules on MSCs appear to play a role in the clearance of administered MSCs from the lung [23]. Umbilical cord MSCs demonstrate a different profile of cell surface markers compared to bone marrow-derived MSCs, and this was implicated in enhanced lung clearance of UC-MSCs. Altering cell surface marker expression on MSCs may be used to decrease—or increase—retention time in the lung, and thereby impact MSC function in vivo.

## Summary and conclusions

The use of MSCs as a therapeutic is a promising, effective strategy in numerous disease and injury states in pre-clinical models. As of 2016, there were 493 MSC-based clinical trials completed or ongoing [120]. Unfortunately, limitations in our understanding of the biodistribution and fate of therapeutically administered MSCs within the body constitute a significant impediment to successful clinical translation of MSCs. The FDA guidelines [121] and the European Medicines Agency [122] guidelines for drug development both require the generation of pre-clinical data on pharmacodynamics and biodistribution before product approval.

While understanding biodistribution and fate in patients is clearly more challenging for a cellular therapy than for a standard pharmacologic, recent advances in imaging techniques now offer the possibility to more systematically develop our knowledge of these issues. A clear characterisation of the in vivo kinetics of MSC therapeutics would provide insight to important unknowns such as the optimal route of administration, the optimal dosage size and regimen, the potential for altering the MSC microenvironment ex vivo to modulate their in vivo kinetics, and indeed whether or not MSCs need to be present at the site of injury.

In summary, a better understanding of patterns of biodistribution and of the fate of MSCs will not only provide much-needed safety data, but will also uncover strategies to increase the distribution and/or persistence of MSC at the sites of injury, potentially increasing their therapeutic potential for multiple disorders in the clinic.

## Abbreviations

AEC: Airway epithelial cell
ARDS: Acute respiratory distress syndrome
BM: Bone marrow
cDNA: Complementary DNA
COX1: Cyclooxygenase-1
CXCR4: CXC chemokine receptor 4
DNIF: Dynamic near-infrared fluorescence
eGFP: Enhanced green fluorescent protein
EMA: European Medicines Agency
FDA: Food and Drug Administration
HLA: Human leukocyte antigen
IA: Intraarticularly
IP: Intraperitoneal
IV: Intravenously
IVIS: In vivo imaging systems
IVM: Intravital microscopy
MRI: Magnetic resonance imaging
MSC: Mesenchymal stromal cell
NIR: Near-infrared
PCR: Polymerase chain reaction
PET: Positron emission technology
SC: Subcutaneous
SPECT: Single photon emission computed tomography
TNF-α: Tumour necrosis factor alpha
UC: Umbilical cord
VCAM: Vascular cell adhesion molecule
